# Intracellular *Mycoplasma genitalium *infection of human vaginal and cervical epithelial cells elicits distinct patterns of inflammatory cytokine secretion and provides a possible survival niche against macrophage-mediated killing

**DOI:** 10.1186/1471-2180-9-139

**Published:** 2009-07-14

**Authors:** Chris L McGowin, Vsevolod L Popov, Richard B Pyles

**Affiliations:** 1 Department of Pathology, University of Texas Medical Branch, 301 University Blvd., Galveston, TX, 77555-0609, USA; 2 Department of Pediatrics, Sealy Center for Vaccine Development, University of Texas Medical Branch, 301 University Blvd., Galveston, TX, 77555-0436, USA

## Abstract

**Background:**

*Mycoplasma genitalium *is an emerging sexually transmitted pathogen that has been associated with significant reproductive tract inflammatory syndromes in women. In addition, the strong association between severity of *M. genitalium *infection and Human Immunodeficiency Virus type 1 (HIV-1) shedding from the cervix suggests that innate responses to *M. genitalium *may influence pathogenesis of other sexually transmitted infections. Epithelial cells (ECs) of the reproductive mucosa are the first cells contacted by sexually transmitted pathogens. Therefore, we first characterized the dynamics of intracellular and extracellular localization and resultant innate immune responses from human vaginal, ecto- and endocervical ECs to *M. genitalium *type strain G37 and a low-pass contemporary isolate, M2300.

**Results:**

Both *M. genitalium *strains rapidly attached to vaginal and cervical ECs by 2 h post-infection (PI). By 3 h PI, *M. genitalium *organisms also were found in intracellular membrane-bound vacuoles of which approximately 60% were adjacent to the nucleus. Egress of *M. genitalium *from infected ECs into the culture supernatant was observed but, after invasion, viable intracellular titers were significantly higher than extracellular titers at 24 and 48 h PI. All of the tested cell types responded by secreting significant levels of pro-inflammatory cytokines and chemokines in a pattern consistent with recruitment and stimulation of monocytes and macrophages. Based on the elaborated cytokines, we next investigated the cellular interaction of *M. genitalium *with human monocyte-derived macrophages and characterized the resultant cytokine responses. Macrophages rapidly phagocytosed *M. genitalium *resulting in a loss of bacterial viability and a potent pro-inflammatory response that included significant secretion of IL-6 and other cytokines associated with enhanced HIV-1 replication. The macrophage-stimulating capacity of *M. genitalium *was independent of bacterial viability but was sensitive to heat denaturation and proteinase-K digestion suggesting that *M. genitalium *protein components are the predominant mediators of inflammation.

**Conclusion:**

Collectively, the data indicated that human genital ECs were susceptible and immunologically responsive to *M. genitalium *infection that likely induced cellular immune responses. Although macrophage phagocytosis was an effective method for *M. genitalium *killing, intracellular localization within vaginal and cervical ECs may provide *M. genitalium *a survival niche and protection from cellular immune responses thereby facilitating the establishment and maintenance of reproductive tract infection.

## Background

*Mycoplasma genitalium *is now recognized as a sexually transmitted pathogen [1,2]. In healthy young men and women, the prevalence of *M. genitalium *infection is approximately 1% and is between those of gonococcal (0.4%) and *Chlamydia trachomatis *(4.2%) infections [2]. In men, *M. genitalium *is a recognized and important cause of non-gonococcal urethritis [3]. Reproductive tract disease syndromes associated with *M. genitalium *infection in women include pelvic inflammatory disease [4] and cervicitis [5-9]. Limited serologic studies and detection of *M. genitalium *DNA in cervical, endometrial and/or Fallopian tube specimens from women with salpingitis [10] have suggested that *M. genitalium *could also be a cause of tubal factor infertility [11,12] independent of *Chlamydia trachomatis*. Importantly, the burden of *M. genitalium *at the cervical mucosa is positively correlated with Human Immunodeficiency Virus type 1 (HIV-1) shedding [13] but the cell types involved and the mechanisms of these associations remain unclear. Select pro-inflammatory cytokines, including IL-6, have been associated with increased HIV-1 titers [14] and up-regulate HIV-1 replication [15]. These findings indicate that *M. genitalium *infection may enhance acquisition or dissemination of other sexually transmitted infections and provide strong rationale for investigation into the host innate immune response.

The mucosal surfaces of the female reproductive tract provide a physical barrier against invading pathogens. Importantly, these surfaces are adapted to constant antigenic stimulation from the normal polymicrobial flora but are concomitantly charged with recognition and response to pathogen exposure. Following sexual transmission, *M. genitalium *and other pathogens make initial contact with epithelial cells (ECs) that play an important role in early activation of the innate response. ECs of the vagina and cervix express robust levels of Toll-like receptor (TLR) 2, 3, 5, 6 and CD14 with low levels of TLR1, 4 and 7–9 [16]. Furthermore, both vaginal and cervical ECs recognize bacterial ligands via TLR2/6 such as the macrophage-activating lipopeptide of *Mycoplasma fermentans *[17]. Although macrophages are not always resident in the vaginal lumen, they are distributed throughout the epithelial and sub-epithelial mucosa of the vagina and cervix and make up a significant proportion of the total immune cell population of the reproductive tract [18]. Generally, macrophages recognize, phagocytose and destroy pathogenic bacteria [19] and studies are needed to address directly the interaction of *M. genitalium *with human macrophages. Specifically, it currently is unclear whether infection of reproductive tract ECs elicits chemokine secretion for recruitment of phagocytic cells to infected tissues resulting in inflammation.

Lipoprotein-enriched detergent phase preparations from *M. genitalium *strain G37 have been reported to activate inflammatory cytokine secretion from a transformed monocytic cell line [20,21] but these fractions have yet to be tested using human genital ECs or cell types more relevant to genital transmission. Recently, our group has shown that human reproductive tract ECs are highly responsive to TLR2/6-activating regions of the MG309-encoded protein resulting in inflammatory cytokine secretion [22]. To further explore the responses of human genital ECs, we have established that *M. genitalium *inoculation of human vaginal, ecto- and endocervical ECs results in both extra- and intracellular infection that elicits tissue-specific cytokine secretion. The elaborated cytokines were consistent with recruitment of macrophages to the reproductive mucosa. In addition, subsequent testing showed that human monocyte-derived macrophages (MDM) rapidly phagocytosed and killed *M. genitalium *resulting in a robust secretion of pro-inflammatory cytokines. These data provide the first characterization of the human innate immune response to viable *M. genitalium *from relevant cell types of the female reproductive tract and provide insight into the dynamic interaction with the reproductive mucosa.

## Methods

### Human cell culture

Immortalized human ECs derived from vaginal (n = 3 donors; V19I, V12I, V11I), ectocervical and endocervical tissues were maintained as described previously [16]. Keratinocyte serum-free medium (KSFM; Invitrogen, Carlsbad, CA) supplemented with bovine pituitary extract (50 mg/L), recombinant epidermal growth factor (5 ug/L), CaCl_2 _(44.1 mg/L), penicillin-G (100 U/mL) and streptomycin sulfate (100 ug/mL) was used for culture of ectocervical and endocervical ECs at 37°C in a 5% CO_2 _humidified incubator [23]. Vaginal ECs were maintained in a 1:1 mixture of KSFM and VEC-100 media (MatTek, Ashland, MA). ME-180 (ATCC HTB-33) cervical carcinoma cells were maintained in RPMI 1640 (MediaTech, Herndon, VA) medium supplemented with 0.1 mM non-essential amino acids (Sigma-Aldrich, St. Louis, MO), 2 mM L-glutamine, penicillin-G (100 U/mL), streptomycin sulfate (100 ug/mL) and 10% fetal bovine serum (FBS; Invitrogen). Cells were verified to be free of any contaminating mycoplasmas by PCR (Stratagene, Cedar Creek, Texas).

### Propagation of *M. genitalium *strains G37 and M2300

*Mycoplasma genitalium *type strain G37 (ATCC 33530) or the more contemporary, lower passage Danish M2300 strain was propagated in Friis FB medium [24]. Briefly, *M. genitalium *stocks (stored at -80°C) were inoculated aseptically into tightly sealed tissue culture flasks containing freshly prepared Friis FB medium and incubated at 37°C for 5–8 d. Growth was monitored by the formation of adherent microcolonies and a pH-mediated color change of the medium. *M. genitalium *was harvested from culture flasks by pouring off the spent medium, extensively washing adherent mycoplasmas with 5 volumes of approximately 5 mL each of sterile PBS and then scraping adherent microcolonies into fresh PBS. *M. genitalium *viability was quantified in 96-well plates by serial 10-fold dilution of each sample into fresh Friis FB medium. The last dilution to show a change in color and formation of microcolonies was used to calculate the approximate number of viable organisms in the original sample. UV-inactivation (254 nm) of *M. genitalium *was performed using a Stratalinker 2400 (Stratagene, La Jolla, CA) to a total energy of 720,000 microjoules/cm^2^. Heat denaturation of *M. genitalium *was accomplished by incubating log-phase cultures at 80°C for 15 min and then rapid cooling on ice. Loss of viability was verified by an absence of growth in Friis FB medium after 14d incubation at 37°C.

### Isolation of human monocyte-derived macrophages

Human macrophages were generated as described previously [25] from peripheral blood mononuclear cells (PBMC) collected from healthy volunteers with University of Texas Medical Branch Institutional Review Board approval. Briefly, PBMCs were isolated using Hypaque-Ficoll (Amersham Biosciences, Piscataway, NJ) density-gradient separation. Selection was performed using the magnetic column separation system (StemCell Technologies, Vancouver, Canada). Purified monocytes were differentiated into macrophages by culturing in RPMI 1640 medium supplemented with 10% FBS, L-glutamine, HEPES, sodium pyruvate and GM- CSF (100 ng/mL). Following 7d of differentiation, monocyte-derived macrophages (MDM) were removed from the culture plastic using a non-enzymatic cell dissociation solution (cat # C1544, Sigma-Aldrich) and then resuspended in fresh RPMI 1640 medium. Macrophage differentiation was verified by flow cytometric confirmation of CD11b, CD80 and CD86 expression showing typical purities of >95% (data not shown). Macrophages were differentiated from PBMCs collected from 3 different blood donors and used in 3 independent experiments.

### Electron Microscopy

#### I. Transmission electron microscopy

Adherent monolayers of *M. genitalium*-inoculated (G37 or M2300; MOI 100) or non-inoculated genital ECs or human MDM (MOI 100) were fixed at indicated times from 2–48 h post-infection (PI) in a mixture of 2.5% formaldehyde and 0.1% glutaraldehyde in 0.05 M cacodylate buffer (pH 7.2) containing 0.03% trinitrophenol and 0.03% CaCl_2_. Cells were scraped, centrifuged briefly at 1,000 × g, washed in 0.1 M cacodylate buffer (pH 7.2) and then post-fixed in 1% OsO_4 _in the same buffer. Each sample was stained *en bloc *with 1% uranyl acetate in 0.1 M maleate buffer, dehydrated in ethanol and embedded in Poly/Bed 812 epoxy resin (Polysciences, Warrington, PA). Ultrathin sections were cut using the Ultracut S ultramicrotome (Reichert-Leica). Sections were stained sequentially in 2% aqueous uranyl acetate and lead citrate and then examined in a Philips 201 or CM 100 electron microscope at 60 kV.

#### II. Scanning electron microscopy

*M. genitalium*-infected and non-infected control cells were fixed as described above for transmission electron microscopy (TEM) for at least 1 h at room temperature, post-fixed in 1% OsO4 in 0.1 M cacodylate buffer, dehydrated in ethanol, treated with hexamethyldisalazane and then air dried. Next, the coverslips were mounted on the specimen stubs and sputter coated with iridium for 40 sec in an Emitech K575X turbo sputter coater (Emitech, Houston, TX). Samples were examined in a Hitachi S4700 field emission scanning electron microscope (Hitachi High Technologies America, Electron Microscope Division, Pleasanton, CA) at 2 kV.

### Quantification of *M. genitalium *uptake by cervical epithelial cells

**Figure 1:**
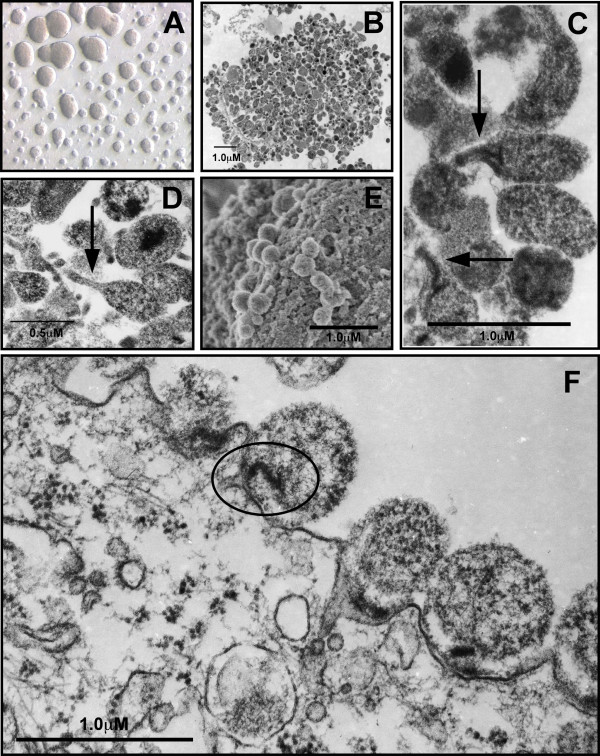
**Cultivation of *M. genitalium *and ultrastructural analysis of attachment to vaginal epithelial cells**. *M. genitalium *G37 or M2300 were grown to log-phase in Friis FB medium. (A) Light micrograph of attached G37 microcolonies grown in culture flasks containing Friis FB medium taken using Variable Relief Contrast (VAREL). (B) TEM micrograph of a single G37 microcolony after 3d growth in Friis FB medium showing highly variable size and morphology. (C) Within *M. genitalium *G37 microcolonies, an elongated tip-like structure (arrow) was observed. (D) TEM micrograph *M. genitalium *strain M2300 showing similar variable morphology compared to G37 and formation of an electron-dense tip structure. Log-phase *M. genitalium *were harvested from Friis medium and then inoculated onto vaginal EC monolayers for ultrastructural analysis of attachment. (E) SEM micrograph of *M. genitalium *G37 attached to vaginal ECs (2 h PI). (F) TEM micrograph of *M. genitalium *G37 attached to vaginal ECs collected 3 h PI. An electron dense core structure presumably involved in attachment and invasion of vaginal ECs is highlighted by the oval. Similar electron dense cores were observed in some tip structures and can be seen in panel C.

Uptake of *M. genitalium *by reproductive tract ECs was assessed using the gentamicin invasion assay [26]. The sensitivity of *M. genitalium *strains G37 and M2300 to gentamicin was established first by inoculation of log-phase organisms into Friis FB medium with gentamicin concentrations ranging from 100–400 ug/mL. No *M. genitalium *growth was observed at 200 or 400 ug/mL therefore a working concentration of 200 ug/mL was employed in subsequent studies to minimize EC uptake of gentamicin and subsequent killing of intracellular *M. genitalium*. Confirmatory studies were completed subsequently using 400 ug/mL gentamicin. As a representative genital EC type, ME-180 cells were seeded into 96-well plates 1d prior to infection at a density of 1 × 10^5 ^cells/well. Log-phase *M. genitalium *was inoculated onto ME-180 cells (MOI of 100) in triplicate. Following 3 h incubation, when *M. genitalium *appeared to be attached to and invading genital ECs (see Figure 1), the inoculum was removed and replaced with fresh medium containing gentamicin. At 15 min, 24 and 48 h following removal of the inoculum, culture supernatants were removed and the infected cells were washed 3× with sterile PBS. Cells then were removed from the well by scraping into Friis FB medium followed by plating serial 10-fold dilutions prepared in Friis FB medium into a 96-well plate. Outgrowth of *M. genitalium *from infected ME-180 cells was observed for 14d. The load of viable *M. genitalium *from each sample was calculated by titration as described above.

The gentamicin invasion assay also was utilized to investigate whether intracellular *M. genitalium *were able to escape from the infected ECs. For these experiments, ME-180 cervical ECs were infected with *M. genitalium *and then extracellular organisms were killed with gentamicin (2 h) after 3 h of infection as described above. Infected ECs then were rinsed 3× with PBS and overlaid with fresh medium that did not contain gentamicin. At 15 min, 24 and 48 h following removal of the gentamicin, culture supernatants were collected for quantification of *M. genitalium *that had egressed from the infected cells. Quantification was performed in triplicate experiments using the CCU assay as described above.

### Stimulation of genital epithelial cells or primary monocyte-derived macrophages

Human vaginal, ectocervical or endocervical ECs were seeded into 96-well plates at a density of 1 × 10^5 ^cells/well. Primary human MDM were seeded into 96-well plates at 5 × 10^4^/well. Following overnight incubation at 37°C, culture supernatants were removed and replaced with fresh medium to remove any constitutively secreted cytokines. Log-phase *M. genitalium *G37 or M2300 was harvested as described above, re-suspended in fresh PBS and then inoculated onto each cell type (MOI of 10). Controls for innate immune stimulation included the *M. salivarium*-derived TLR2/6 agonist, FSL-1 (0.1 ug/well) or an equal volume of the PBS vehicle added to triplicate wells and processed in parallel. Secreted cytokines were quantified from culture supernatants 6 or 48 h PI via a cytometric bead array (CBA) assay using the human 27-Plex panel of cytokine targets (Bio-Rad Laboratories, Hercules, CA). For testing of *M. genitalium *viability following macrophage exposure, infected macrophages were inoculated into Friis FB medium 30 min, 2, 6 or 12 h PI and observed for *M. genitalium *outgrowth indicated by a pH-mediated color change and adherent microcolony formation.

### Statistical Analyses

The Student's t test was used to calculate significant differences in intra- and extracellular *M. genitalium *titers and when comparing secretion of individual cytokines from a single cell type to basal (PBS-treated) levels. The one-way ANOVA followed by Dunnett's post-test (Prism v. 4.0, GraphPad, San Diego, CA) was used to calculate significant differences in cytokine secretion levels when more than 2 conditions were compared. Significance was indicated when p < 0.05.

## Results

### *M. genitalium *ultrastructure, attachment and invasion of human genital epithelial cells

*M. genitalium *strain G37 or M2300 grown to log phase in Friis medium resulted in adherent microcolony formation and were characterized by a radial gradient of colony diameter (Figure 1A). Within each microcolony, *M. genitalium *organisms were densely packed and highly pleomorphic (Figure 1B). Several organisms were observed that showed a tip-like structure (noted with arrows) for both the Danish M2300 strain (Figure 1C) and G37 (Figure 1D).

**Figure 2:**
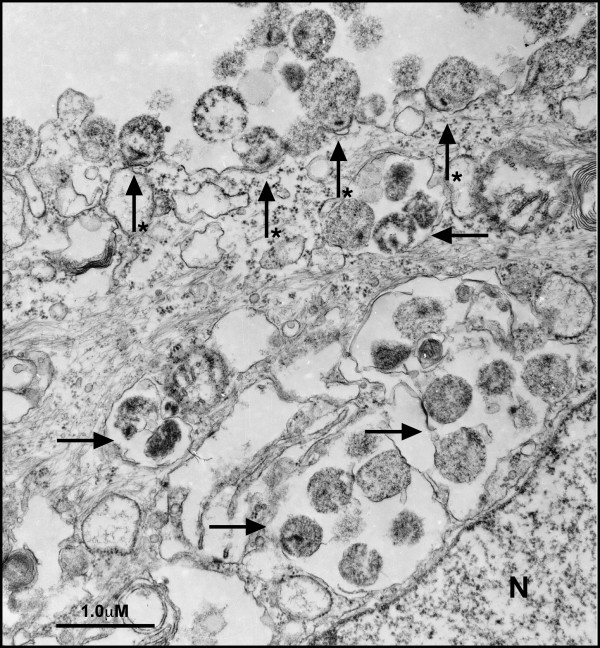
**Attachment and invasion of vaginal epithelial cells by *M. genitalium***. *M. genitalium *G37 or M2300 were harvested from log-phase cultures in Friis FB medium and then inoculated onto vaginal ECs. After 3 h of infection, cells were fixed and processed for TEM imaging. Many *M. genitalium *organisms were attached to the host cell surface associated with a polarized electron-dense core structure (starred arrow). In addition, *M. genitalium *organisms were localized to intracellular vacuoles (arrows) distributed throughout the cellular cytosol. Approximately 60% of observed vaginal ECs showed intracellular vacuoles directly adjacent to the nucleus (denoted as N). Similar findings were observed in cervical ECs and for the Danish M2300 strain.

*M. genitalium *has been shown previously to occupy intracellular spaces in cultured cells of non-reproductive origin [27-29] and cells obtained clinically from vaginal swabs of *M. genitalium*-positive women [30]. However, the invasion dynamics for genital cell types are not well understood. Inoculation of genital ECs with *M. genitalium *strains G37 or M2300 (MOI 100 for electron microscopy) resulted in attachment to vaginal (V19I; Fig 1E) and cervical (ME-180; data not shown) ECs by 2 h PI. Attachment of *M. genitalium *G37 and M2300 to reproductive tract ECs was consistently characterized by a polarized electron-dense core, within the *M. genitalium *organism [31], seen adjacent to the host cell membrane (core indicated in Figure 1F). This dense core was evident within some tip structures as shown for M2300 (Figure 1C). After 3 h infection, *M. genitalium *G37 were attached to the host cells (Figure 2; starred arrows) and also observed in intracellular vacuoles distributed throughout the cellular cytosol (Figure 2; arrows). In approximately 60% of examined cells, intracellular vacuoles were directly adjacent to the nucleus (N; Figure 2). Similar findings were observed 6–48 h PI (data not shown) for both the G37 and M2300 strain. At these later time points, extracellular *M. genitalium *also were observed but were often in aggregates and showed no evidence of attachment or invasion of host cells. Morphologically, the intracellular and extracellular mycoplasmas were highly pleomorphic and appeared to have normal ultrastructure indicated by a dense content of ribosomes and few degraded bacterial membranes. A previously described tip structure [27] was observed readily on *M. genitalium *grown in Friis FB medium (Figure 1C and 1D) but an elongated tip structure was not always visible on mycoplasmas attached to host cells in each stained section. No similar organisms or structures were observed in non-infected cells processed in parallel.

**Figure 3:**
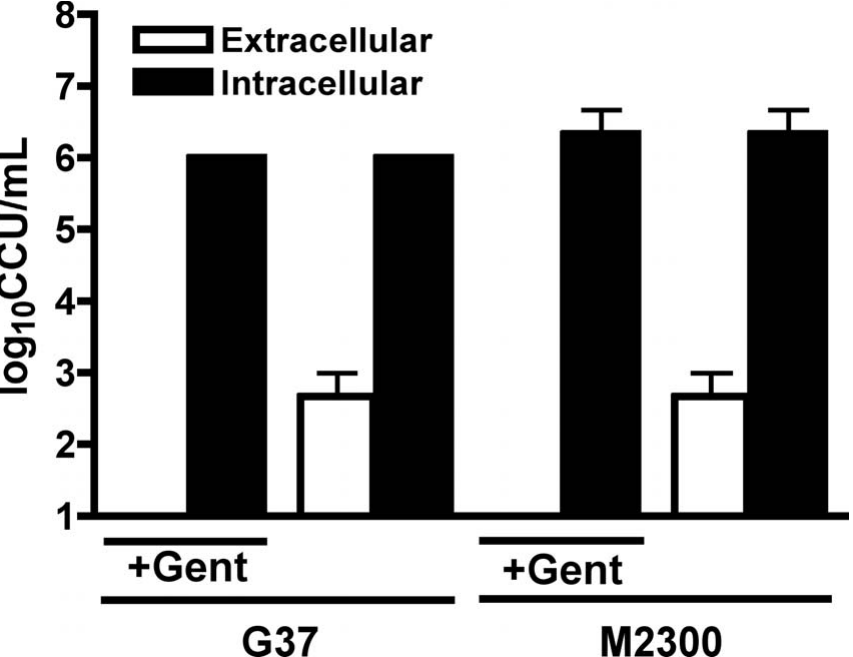
**Intra- and extracellular localization of *M. genitalium *in ME-180 cervical epithelial cells**. Cervical ECs (ME-180) were inoculated with log-phase cultures of *M. genitalium *strain G37 (A) or M2300 (B) to determine whether *M. genitalium *can invade human reproductive tract ECs. To quantify intracellular *M. genitalium *loads (solid bar), the inoculum was removed following 3 h incubation for attachment and entry and replaced with medium containing gentamicin (200 ug/mL). The ability for *M. genitalium *to escape infected ECs (open bar) was quantified from culture supernatants in separate wells processed the same way except, following the 3 h incubation, the inoculum was removed and extracellular *M. genitalium *organisms were killed with gentamicin (2 h exposure). Infected cells then were washed thoroughly and received fresh medium without gentamicin allowing escaping *M. genitalium *to survive. Cell fractions or culture supernatants were collected at 48 h following removal of the inoculum for quantification of bacterial loads using a color changing unit (CCU) assay. In every case, significant differences between intracellular and extracellular *M. genitalium *titers were observed (p < 0.05; Student's t-test). Parallel studies were performed that employed 400 ug/mL gentamicin with similar results (data not shown).

We next quantified *M. genitalium *G37 and M2300 viability from intra- and extracellular fractions of cultured ME-180 cells using a gentamicin protection assay as described in the *Methods*. To quantify intracellular titers, the *M. genitalium *inoculum was incubated for 3 h to allow attachment to and entry of host cells (See Figure 1) followed by removal of the inoculum and replacement of fresh culture medium containing a bactericidal concentration of gentamicin (200 ug/mL). Fifteen minutes following inoculation with either G37 or M2300 (Figure 3), no viable *M. genitalium *were detected in the cells (data not shown). Using a color changing unit assay (CCU), high titers of viable intracellular *M. genitalium *were detected at both 24 h (not shown) and 48 h PI (Figure 3). No *M. genitalium *viability was detected in supernatants containing gentamicin at either point indicating that the observed titers were due exclusively to intracellular mycoplasmas that were protected from gentamicin exposure. Extracellular *M. genitalium *titers, representing organisms that had escaped from infected cells, were quantified from separate wells using supernatants of infected cells. Extracellular titers from culture supernatants (dashed line) were significantly less than intracellular titers (p < 0.05) at the tested time points (48 h shown in Figure 3). These data indicated that, after *M. genitalium *entry of the cell, more organisms remained inside the cell than egressed to the culture supernatant. Intracellular localization of *M. genitalium *in vaginal and cervical ECs also was consistent with electron microscopic analyses (Figure 1 and 2).

### *M. genitalium *elicited pro-inflammatory cytokines from genital epithelial cells

Following demonstration of intracellular localization within reproductive tract ECs, we evaluated the host cytokine response from 3 human vaginal (V11I, V12I, and V19I) and 2 cervical EC lines (sA2EN and 3ECI) [16]. Of the tested time points, peak cytokine values were obtained 48 h PI and are presented in Table 1. Vaginal ECs exposed to viable *M. genitalium *G37 or M2300 (MOI 10) responded with significant secretion of interleukin-6 (IL-6), IL-8 and GM-CSF (p < 0.05 vs. PBS control). Following *M. genitalium *exposure, ectocervical ECs secreted significant levels of IL-6 and IL-8 (p < 0.05 vs. PBS control). Endocervical ECs responded to *M. genitalium *with the most number of secreted cytokines that included IL-6, IL-8, G-CSF, GM-CSF and MCP-1 (p < 0.05 vs. PBS control). Using IL-8 secretion at 48 h PI as a comparator for all cell types, endocervical ECs were more responsive than vaginal or ectocervical cells when the fold increase of cytokine secretion by infected cells was calculated and compared to cells that received only PBS (ANOVA; p < 0.01, data not shown). A similar pattern of cytokine elaboration was observed following inoculation of *M. genitalium *at a MOI of 1 (data not shown). Cytokines that were not significantly induced by *M. genitalium *G37 or M2300 in any genital EC type included IL1-b, IL-2, IL-4, IL-5, IL-7, IL-9, IL-10, IL-12(p40), IL-12(p70), IL-13, IL-15, IL-17, MIP1-a, MIP1-b, Basic FGF, Eotaxin, IP-10, PDGF-BB and VEG-F. The pattern of cytokines elaborated from cervical ECs was consistent with monocyte and macrophage recruitment and thus we next evaluated the responses of primary human MDM to *M. genitalium *exposure and determined whether these cells were capable of *M. genitalium *phagocytosis and killing.

**Table 1:** Cytokine elaboration from human genital epithelial cells following *M. genitalium *G37 exposure^*a*^.

	Vaginal (V19I, V12I, V11I)		Ectocervical (3ECI)		Endocervical (sA2EN)	
	**MOI 10**	**PBS**	**MOI 10**	**PBS**	**MOI 10**	**PBS**

IL-6	127 ± 13.1*	69 ± 1.7	63.7 ± 1.8*	21.3 ± 2.4	348 ± 13*	196 ± 15
IL-8	1458 ± 117*	785 ± 11.3	3304 ± 300*	722 ± 98	5e7 ± 1347*	6e4 ± 367
G-CSF	261 ± 46	227 ± 37	548 ± 143	779 ± 122	155 ± 6.2*	93 ± 21
GM-CSF	24 ± 1.8*	8 ± 3.1	16 ± 2.6	10 ± 1.0	160 ± 9.4*	45 ± 12
MCP-1	5.8 ± 1.4	7 ± 2.1	11.4 ± 1.3	10 ± 3.1	7.2 ± 1.1*	0.46 ± 0.02

^*a*^Human vaginal (n = 3 donors), ectocervical or endocervical ECs were inoculated with *M. genitalium *G37 (MOI 10). An equal volume of the PBS vehicle was added and processed in parallel as a control. Culture supernatants were collected 48 h PI to quantify secreted cytokines. Values are expressed as the mean ± SEM pg/mL from triplicate wells. Cytokine elaboration pattern and magnitudes induced following exposure to strain M2300 were not significantly different than G37. PBS values are presented to indicate basal cytokine elaboration from each cell type. ND, not detected. *, *p *< 0.05 vs. PBS control using Student's t-test.

### Phagocytosis of *M. genitalium *by human monocyte-derived macrophages

**Figure 4:**
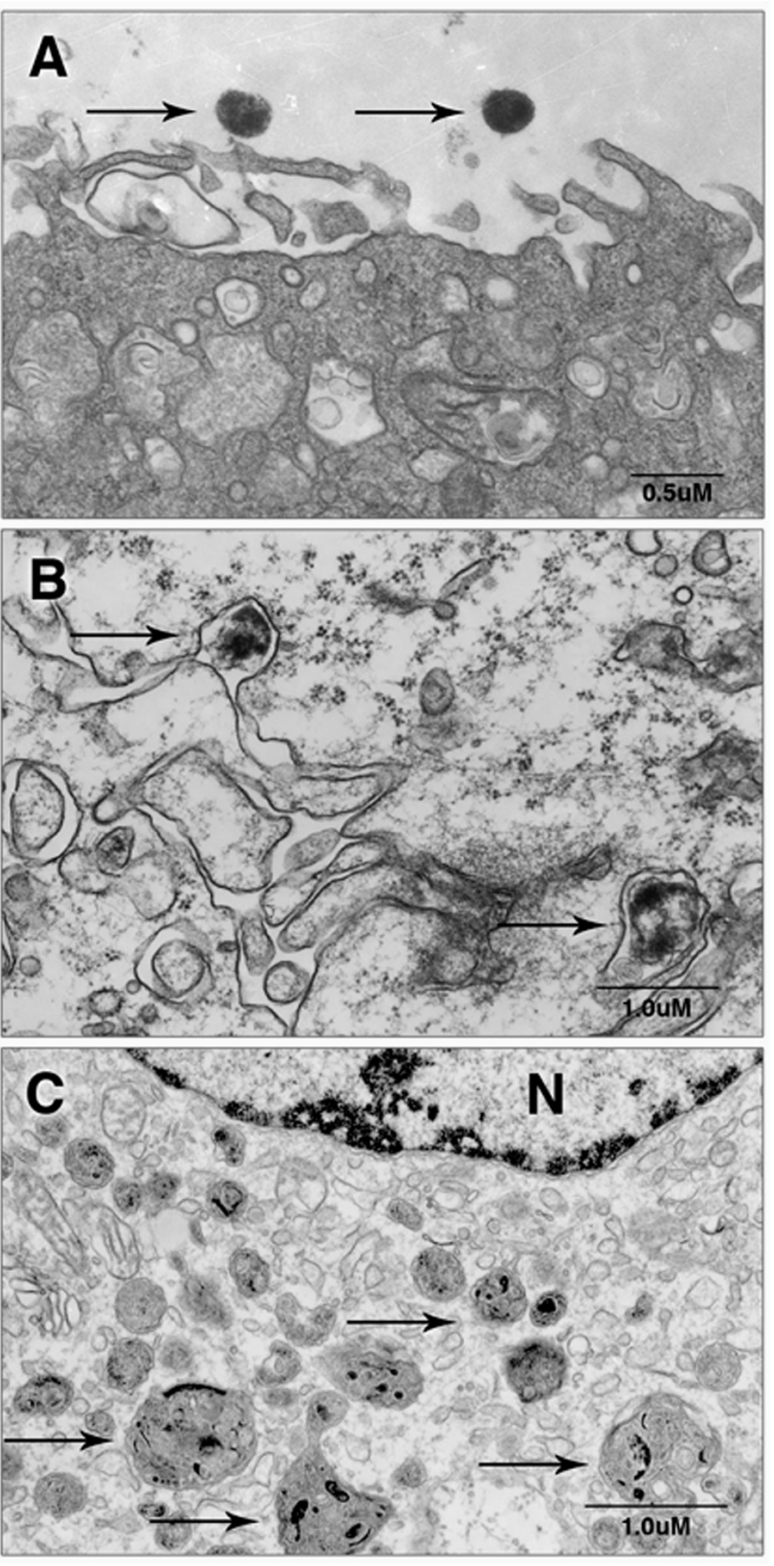
***M. genitalium *was phagocytosed rapidly by human monocyte-derived macrophages resulting in a loss of bacterial viability**. Primary human MDM were inoculated with log-phase *M. genitalium *G37 or M2300 (MOI 100) and collected just after inoculation or 30 min or 6 h PI and processed for TEM. Viable extracellular *M. genitalium *with dense intracellular ribosomes and an intact outer membrane were observed at the time of inoculation (A). Thirty minutes following inoculation, phagocytosis of *M. genitalium *was observed with localization to phagolysosomes (arrow) and morphological changes of the bacterium (B). By 6 h PI, macrophages contained many phagocytic vacuoles (arrows) and no intracellular mycoplasmas could be located (C). Micrographs depict *M. genitalium *strain G37 but similar findings were observed for strain M2300. N denotes nucleus.

To determine the susceptibility of *M. genitalium *to macrophage phagocytosis, human MDM were exposed to log-phase *M. genitalium *strains G37 or M2300 (MOI 100) and processed at selected time points for TEM. Within 5 min of inoculation, *M. genitalium *appeared dark staining with a dense content of ribosomes and no signs of membrane degeneration (Figure 4A). As early as 30 min PI, *M. genitalium *uptake by MDM was observed that was associated with morphologic changes of the bacterium including a loss of ribosome density and a hollow appearance (Figure 4B) consistent with a loss of viability. Internalized *M. genitalium *were prevalent and localized to membrane-bound phagolysosomes. Similar morphological changes were observed 2 h PI (data not shown). By 6 h PI, the macrophages appeared to have many phagocytic vesicles but no intracellular organisms could be located (Figure 4C). Viability of *M. genitalium *following macrophage exposure was evaluated by seeding infected MDM (6 h PI) into Friis FB medium at 37°C. These cultures were observed for *M. genitalium *outgrowth by a pH-mediated color change and microcolony formation. No growth was detected over a 14d period from any of these cultures collectively indicating that *M. genitalium *was susceptible to rapid phagocytosis and killing.

### *M. genitalium *elicited pro-inflammatory cytokines from human monocyte-derived macrophages

**Figure 5:**
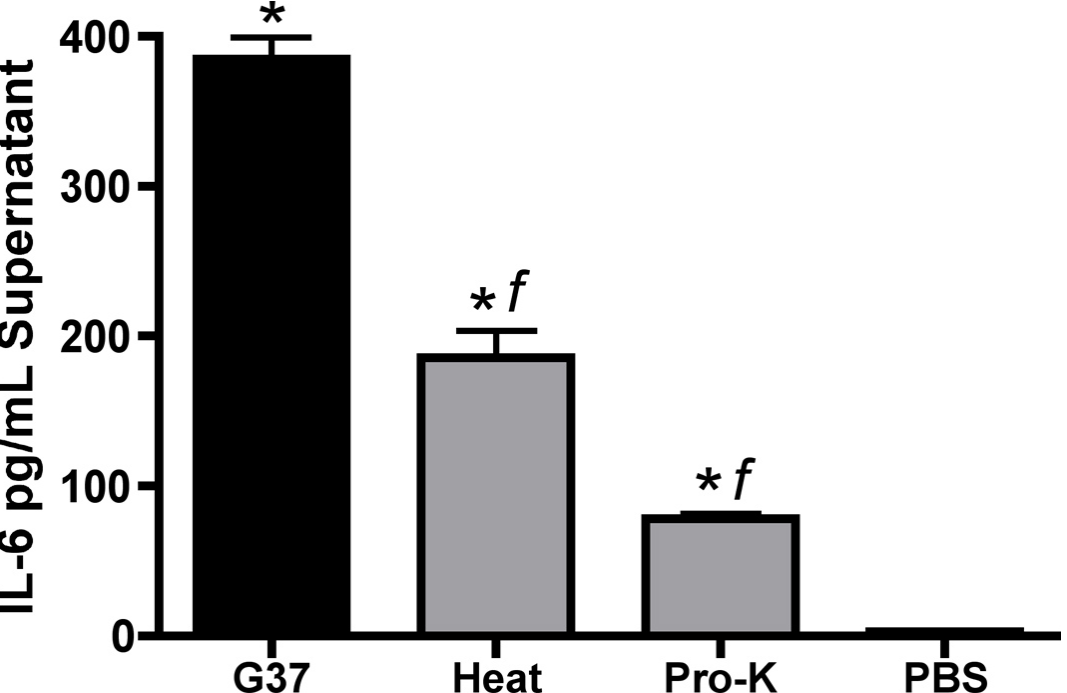
**The *M. genitalium*-induced inflammatory cytokine secretion from human monocyte-derived macrophages was mediated predominately by proteins**. Human MDM were exposed to viable *M. genitalium *G37 (MOI 10) or viable *M. genitalium *that had been denatured by heat or digested with proteinase-K (MOI 10). Cytokine secretion was quantified from culture supernatants collected 6 h following exposure as described in the *Methods*. Data shown are the mean ± SEM of IL-6 (a representative acute-phase cytokine) induction from a typical experiment using *M. genitalium *strain G37 performed in triplicate wells compared to vehicle control (PBS) wells analyzed in parallel for each cell type. Data collected for each experiment using strain G37 or M2300 were similar in pattern and magnitude between 2 additional blood donors. *, p < 0.01 vs. PBS control using ANOVA. *f*, p < 0.01 vs. viable *M. genitalium *G37.

Because *M. genitalium *was phagocytosed rapidly by human MDM with no evidence of bacterial viability by 6 h PI, we sought to determine whether *M. genitalium *exposure to human MDM would elicit acute-phase cytokine responses. Viable *M. genitalium *G37 and M2300 inoculated at MOI 10 or MOI 1 elicited significant cytokine elaboration from macrophage cultures measured from supernatants collected 6 h PI (G37 [MOI 10] results presented in Table 2). No significant differences were observed between G37 and M2300 (data not shown). The profile of induced cytokine responses from human macrophages was composed predominately of early pro-inflammatory markers including significant secretion of IL-1β, TNF-α, IL-6, IL-8, G-CSF, IFN-γ, MCP-1, MIP-1α, MIP-1β and RANTES (p < 0.05; Table 2). These findings were consistent with results from 2 additional blood donors (data not shown). Following UV inactivation, *M. genitalium *elicited a similar profile and magnitude of cytokine secretion (Table 2) indicating that the immunostimulatory capacity was not dependent upon bacterial viability. Immune markers that were not induced by *M. genitalium *in human MDM included IL-2, IL-4, IL-5, IL-7, IL-9, IL-12(p70), IL-13, IL-15, IL-17, Basic FGF, Eotaxin, IP-10, PDGF-BB and VEG-F. Considering IL-6, a representative acute-phase cytokine with a central role in innate responses to bacterial pathogens [32], heat denaturation or proteinase-K digestion of *M. genitalium *significantly reduced the inflammatory capacity from human macrophages (Figure 5) suggesting that a significant proportion of the inflammatory capacity was mediated by *M. genitalium *protein components.

**Table 2:** Cytokine elaboration from human monocyte-derived macrophages following exposure to *M. genitalium *G37^*a*^.

Human MDM			
	**Viable**	**UV-Inactivated**	**PBS**

**IL-1β**	31 ± 6.1*	33 ± 1.4*	0.7 ± 0.04
**IL-6**	385 ± 13.8*	439 ± 4.0*	3.2 ± 0.1
**IL-8**	5784 ± 149*	5368 ± 564*	116 ± 7.8
**G-CSF**	63.1 ± 5.5*	72 ± 2.4*	6.2 ± 0.1
**IFN-γ**	270 ± 24*	339 ± 3.9*	9 ± 3.6
**MCP-1**	298 ± 9.3*	318 ± 8.3*	36 ± 3.9
**MIP-1α**	1056 ± 16*	1068 ± 4.0*	176 ± 10.9
**MIP-1β**	2514 ± 57*	2403 ± 19*	810 ± 47
**RANTES**	66 ± 1.5*	74 ± 9.9*	11.4 ± 0.4
**TNF-α**	7456 ± 334*	8616 ± 697*	20 ± 2.0

^*a *^Human MDM were inoculated with *M. genitalium *G37 (MOI 10) or an equal volume of the PBS vehicle as a control into triplicate wells. Culture supernatants were collected 6 h PI to quantify secreted cytokines as described in the *Methods*. Values are expressed as the mean ± SEM pg/mL supernatant. PBS values are presented to indicate basal cytokine elaboration. Data collected following exposure to *M. genitalium *strain M2300 were similar in pattern and magnitude. ND, not detected. *, p < 0.01 using ANOVA.

## Discussion

Considering that *M. genitalium *reproductive tract infections in humans [1,33] and non-human primates [34] are often persistent, it seems likely that *M. genitalium *employs some tactic(s) to elude the host response to establish infection. Consistent with this hypothesis, attachment to and invasion of vaginal and cervical ECs by *M. genitalium *strains G37 and M2300 was observed by a subset of organisms as early as 2 h PI (Figure 1) suggesting that intracellular localization could provide a survival niche. The intracellular *M. genitalium *organisms were found in intracellular vacuoles similar in appearance to those seen in cultured Vero [27], HeLa and EM42 cells [35]. Approximately 60% of *M. genitalium*-containing vacuoles were adjacent to the nucleus but also were distributed throughout the cytoplasm similar to a previous observation in cultured human endometrial cells [35]. Considering more than 20 h of microscope time and over 30 examined grids, it was concluded that more than 95% of cells showed attached *M. genitalium *organisms with roughly 50% of cells containing intracellular vacuoles with *M. genitalium *collected 0–48 h PI. Importantly, no *M. genitalium *organisms were ever observed free in the cytosol but were always bounded by a vacuolar membrane.

Our findings are the first report of intracellular localization in cultured human ECs from the vagina, ecto- and endocervix. These cell types are likely the first target cells following sexual transmission and therefore acute-phase interaction and host response are vital to understanding how *M. genitalium *establishes reproductive tract infection. The observation of *M. genitalium *invasion of vaginal and cervical ECs (Figure 1 and 2) is consistent with the clinical observation of heavy intracellular *M. genitalium *loads in PCR-positive vaginal specimens [30] and is substantiated by earlier reports of intracellular localization in cells of non-reproductive tract origin [27-30]. From our gentamicin invasion studies, *M. genitalium *was found both at intracellular sites and in extracellular fractions of infected cells. These outcomes were consistent with our electron microscopy studies as well. However, additional investigation will be required to address intracellular *M. genitalium *replication within host reproductive tract ECs as the experimental systems utilized for our studies did not facilitate reliable quantification of this outcome. Interestingly, it also was observed that, following intracellular localization by *M. genitalium*, a low level of egress from infected cells occurred (Figure 3) from 5–48 h PI suggesting that periodic egress from infected cells could result in cell to cell spread. Collectively, these results firmly indicate *M. genitalium*'s capacity for invasion and prolonged intracellular survival that could provide the organism with a long-term survival niche in reproductive tract tissues.

From our studies of vaginal and cervical ECs, *M. genitalium *was observed at both intracellular and extracellular sites. However, it is not clear whether the invasive organisms are genetically different than those that were observed outside of the cells or whether some unknown factor facilitates entry of some organisms while excluding others. In addition, a well-defined tip structure [27,31] was rarely observed in our studies despite robust attachment to and invasion of the vaginal and cervical ECs (Figure 1 and 2) used in these studies. An area of increased electron density was observed within the *M. genitalium *organism (Figure 1C, F and 2) adjacent to the host cell surface presumably involved in attachment to the host cell. A similar structure has been described previously for *M. genitalium *strains grown attached to plastic cultureware [31]. These phenomena suggest that *M. genitalium *attachment to and invasion of reproductive tract ECs may not require a well-defined tip structure. In addition, attachment and invasion may involve cellular receptors that are localized to specific membrane sites that are better modeled using polarized 3-dimensional EC cultures. Indeed, the observed egress of *M. genitalium *from infected mucosal ECs likely would lead to infection of an adjacent cells in vivo rather than into the culture supernatant of traditional 2-dimensional cultures. This considered, a 3-dimensional multi-layer model of vaginal EC infection might better address how *M. genitalium *interacts with the host mucosa and establishes primary reproductive tract infection.

Because ECs likely serve as the first responders to STI, we investigated the acute-phase cytokine response to *M. genitalium *from human vaginal and cervical ECs. We found that *M. genitalium *elicited minimal innate responses from human vaginal ECs from 3 donors but ecto- and endocervical ECs were highly responsive and secreted cytokines consistent with recruitment of immune cells including IL-8, G-CSF, GM-CSF and MCP-1 (Table 1). The increased responsiveness of endocervical ECs may have biological relevance, as the normally sterile upper tract tissues likely are more sensitive to microbial contamination than the lower genital tract. Paradoxically, it is in the upper tract tissues where inflammation due to microbial infection likely has the most severe consequences potentially leading to PID, salpingitis or reduced fertility [36]. Our studies were focused primarily on the lower genital tract but the heightened sensitivity of endocervical ECs provides rationale for testing cell types of the upper tract including endometrial [35] and fallopian ECs. All of the cell types used for cytokine analysis were immortalized by transduction of the human papilloma virus E6/E7 genes known to reduce the levels, but preserve the pattern of cytokine secretion relative to primary progenitor cells [16]. Therefore, we are confident that the observed cytokine inductions indicate the character of the responses but likely underestimate the actual levels of secretion.

Considering the profile of secreted cytokines by *M. genitalium*-infected reproductive ECs, we next investigated whether macrophages could play a role in the cellular immune response to *M. genitalium*. Following exposure to human MDM, phagocytosis of *M. genitalium *occurred rapidly (Figure 3) resulting in complete ablation of bacterial viability by 6 h PI. Importantly, several key pro-inflammatory cytokines were induced in response to *M. genitalium *exposure. IL-6 secretion may be of particular importance considering that IL-6 from vaginal secretions is positively correlated with HIV-1 burden [14] and known to up-regulate HIV-1 replication [15]. Indeed, the microbial burden of *M. genitalium *in clinically-obtained cervical specimens also is strongly associated with HIV-1 shedding [13]. Furthermore, macrophages are one of two major cellular reservoirs for latent HIV-1 infection and contribute to early-stage virus transmission and dissemination throughout the host (reviewed in [37]). To this end, we observed significant secretion of 4 potent chemokines responsible for granulocyte recruitment, MIP1-a, MIP1-b [38], MCP-1 and RANTES [39] (Table 2) indicating that macrophage exposure to *M. genitalium *in reproductive tissues likely would result in significant inflammation consistent with enhanced HIV-1 replication. Our findings suggest that both infected genital ECs and recruited immune cells are responsible for secretion of IL-6 and other cytokines that may contribute to HIV-1 pathogenesis but continued research is necessary to dissect the cellular dynamics of HIV-1 and *M. genitalium *co-infections.

In our studies, the macrophage-stimulatory capacity of *M. genitalium *was not dependent upon bacterial viability. This outcome likely is due to the highly sensitive nature of macrophages. However, both heat denaturation and proteinase-K digestion significantly reduced the cytokine response (Figure 5) suggesting that a large proportion of *M. genitalium*'s inflammatory capacity is indeed mediated by protein components. In addition, other findings from our group showed that *M. genitalium *and the antigenic MG309-encoded protein activate TLR2/6 to induce pro-inflammatory cytokine secretion from human MDM and reproductive tract ECs [22]. Collectively, these results indicated that macrophages are highly sensitive to *M. genitalium *exposure and highlight the putative pressure to evade the cellular immune responses.

Establishment of primary infection and persistence by *M. genitalium *in host tissues is not well understood. Our findings suggest that a subset of *M. genitalium *organisms rapidly invade host ECs thereby exploiting an intracellular survival niche to evade the potent and effective cellular host immune responses. Studies that address directly whether reproductive ECs provide protection from macrophage phagocytosis are currently underway and will be essential to understand this mechanism of immune evasion. Importantly, *M. genitalium *infection resulted in acute-phase inflammatory cytokine responses from vaginal and cervical ECs. Therefore, it is possible that persistent infection of female reproductive tract tissues may indeed result in inflammatory outcomes that could affect reproductive health but continued research is necessary to fully elucidate the mechanisms of *M. genitalium*-induced urogenital disease in women.

## Conclusion

Human vaginal, ecto- and endocervical ECs were susceptible to *M. genitalium *G37 and M2300 infection resulting in rapid intracellular localization of a subset of organisms and significant secretion of pro-inflammatory cytokines. The pattern of cytokine secretion was consistent with recruitment and stimulation of monocytes and macrophages at the vaginal and cervical mucosa. Following exposure to human monocyte-derived macrophages, *M. genitalium *was killed rapidly and elicited a potent pro-inflammatory response including secretion of cytokines associated with enhanced HIV-1 replication. These are the first data showing that cultured human vaginal and cervical ECs are susceptible and immunologically responsive to *M. genitalium *infection likely inducing cellular immune responses to infected tissues. Continued investigation of whether intracellular localization in reproductive tract ECs provides protection from the cellular immune response is warranted but rapid invasion of vaginal ECs, combined with the low immunological response, provides evidence for how *M. genitalium *might efficiently establish reproductive tract infection.

## Authors' contributions

CLM carried out the intracellular dynamic studies, cytokine quantification assays, electron microscopy and drafted the manuscript. VLP provided assistance and direction in the study design and sample processing for electron microscopy. RBP participated in the study design, directed the overall research and helped draft the manuscript. All authors read and approved the final manuscript.
